# Management of Enteroatmospheric Fistula (EAF) Using a Fistula-Vacuum Assisted Closure (VAC) in a Complicated Abdominal Trauma Case

**DOI:** 10.7759/cureus.37668

**Published:** 2023-04-17

**Authors:** Connor J English, Oluwafolaranmi E Sodade, Cindy L Austin, Jason L Hall, Brian B Draper

**Affiliations:** 1 Trauma Surgery, A.T. Still University - Kirksville College of Osteopathic Medicine, Kirksville, USA; 2 Trauma Research, Mercy Hospital, Springfield, MO, USA; 3 Trauma Surgery, Mercy Hospital, Springfield, MO, USA

**Keywords:** enterocutaneous fistula, fistula-vac, enteroatmospheric fistula, endoscopic retrogade and cholangiopancreatography, exploratory laparatomy

## Abstract

Enteroatmospheric fistula (EAF) is a relatively rare complication of patients undergoing open abdomen (OA) for damage control surgery. Mortality rates are high due to the increased risk of peritonitis, intraabdominal abscess, sepsis, and new perforations. There are a wide range of EAF management therapies in the literature, however, there are limited options on cases involving fistula-vaccum assisted closure (VAC) therapy. This case describes the treatment course of a 57-year-old, male admitted for blunt abdominal trauma secondary to a motor vehicle accident. Upon admission the patient underwent damage control surgery. The surgeons elected to have the patient's abdomen open, applying a mesh to promote healing. After several weeks of hospitalization an EAF was discovered in the abdominal wound subsequently managed by utilizing a fistula-VAC technique. Based on the successful outcome of this patient, fistula-VAC was shown as an effective way to promote wound healing while reducing the chances of complications.

## Introduction

Enteratmospheric fistula (EAF) is one of the most feared complications of an open abdomen (OA), presenting a complex set of surgical, nutritional, and critical care challenges [1]. OA is a popular cornerstone of damage control surgery, with indications that include trauma, abdominal sepsis, abdominal compartment syndrome, and loss of abdominal wall [2]. EAF is akin to enterocutaneous fistula; an abnormal tract between the gastrointestinal tract and the skin. Older literature often does not make the distinction between the two [3]. However, in EAF, the intestinal tract is open directly to the atmosphere, without any cutaneous or subcutaneous covering [4]. This defect allows for spillage of intestinal contents directly into the peritoneal cavity and onto surrounding viscera, increasing the risk of sepsis, abscess formation, and new perforations [5-6]. EAF is a rare condition, with an estimated incidence of 7%-12% of patients undergoing OA in the setting of damage control surgery [7-9]. The mortality risk associated with EAF is approximately 40% [10]. Spontaneous resolution of such fistulae is rare, and management is often complicated by granulation tissue and peritoneal adhesions, or “frozen abdomen” [11]. Management of EAF often involves completely diverting the fistula in a way that allows for controlled collection of contents, effectively converting the defect into a chronic, manageable ostomy [2]. Numerous methods are presented in the literature for both, isolation of the fistula, and management of the OA. Some popular techniques include floating stoma [12], variations of a fistula vacuum-assisted closure (VAC) [13], and variations of a baby bottle nipple VAC [14]. Currently there is no consensus standard of care. Management is unique to each patient situation and each surgeon’s level of experience and comfort with a given treatment approach [1]. We present a case describing the management of an EAF in a patient with OA following damage control surgery for blunt abdominal trauma.

## Case presentation

**Figure 1:**
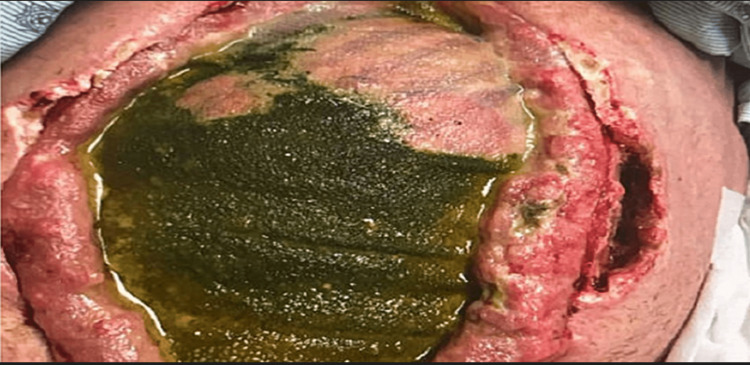
Bowel content contamination through abdominal wall mesh.

The patient is a 57-year-old male, brought to the trauma center via Emergency Medical Services following a motor vehicle accident. Upon initial assessment, Glasgow Coma Scale was three, poly-trauma was identified which included multiple rib fractures with flail chest, bilateral pneumothoraces, and obvious facial fractures, among other injuries. He had a positive Focused Assessment with Sonography in Trauma (FAST) exam with hemorrhagic shock, hemoglobin 10.2 g/dL, 33.6% hematocrit level, and a shock index value of 2.0 in the trauma bay. Massive transfusion protocol was initiated, and the patient was promptly taken to the operating room (OR) for exploratory laparotomy. During the initial procedure the patient underwent splenectomy for Grade IV splenic laceration, partial right non-anatomic hepatectomy for Grade V liver laceration, and intra-abdominal packing. The abdomen was left open and an ABTHERA™ wound VAC (3M, St. Paul, MN) was applied. On hospital day two, the patient returned to the OR for ileocecectomy, repair of a left hepatic duct laceration, and repacking. On day three, extended partial hemi-colectomy, ileocolic anastomosis, and facial closure were performed. The patient underwent open reduction and internal fixation of a right femoral neck fracture on hospital day five. On hospital day eight, the patient became febrile with body temperature, 100.6°F, leukocytosis (white blood cells 25.1 K/uL, lymphocytes 3%) and returned to the OR for exploratory laparotomy and washout. Distal pancreatectomy and omentectomy were performed at this time. On day 10, component separation and closure with Phasix™ mesh (BD, Franklin Lakes, NJ) was performed. The patient progressed slowly over the next several weeks with the only significant event being an endoscopic retrograde cholangiopancreatography (ERCP) with stent placement, for treatment of a moderate common hepatic duct bile leak on day 24 of the admission. Several days after the ERCP, the patient became febrile with marked leukocytosis. Stool was appreciated beneath and surrounding the Phasix™ mesh, and anastomotic breakdown was suspected (see Figure 1).

**Figure 2:**
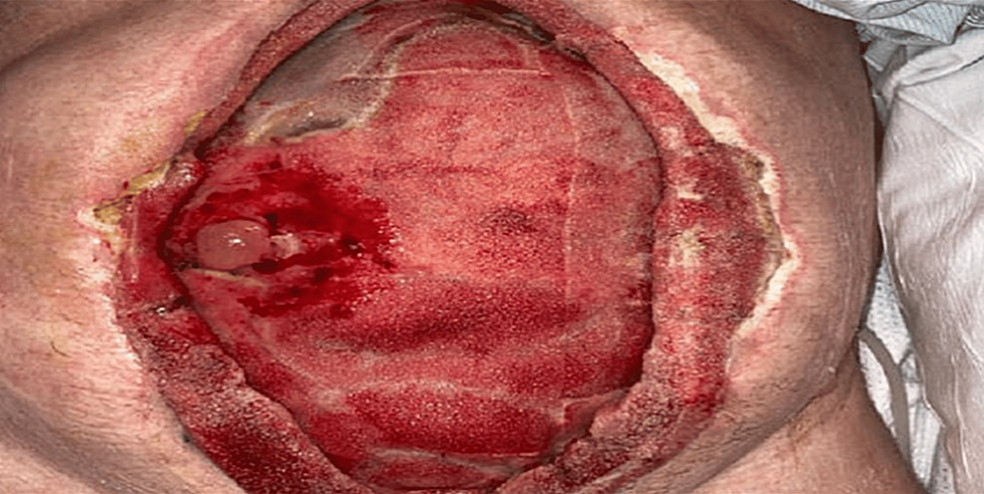
Irrigated abdominal wall wound with granulated bowel and fistula/stoma located at 9 o’clock position. Approximate wound dimensions: 25 cm x 15 cm.

The patient returned to the OR on hospital day 30 for mesh removal and exploration. As expected, the bowel had undergone significant granulation under the mesh and repeat laparotomy was not considered. There was evidence of an anastomotic breakdown with development of an EAF causing bowel content leakage on the right lateral margin of the dehiscent abdominal wound. The mesh was removed, and the granulated bowel was thoroughly irrigated of stool (see Figure 2).

**Figure 3:**
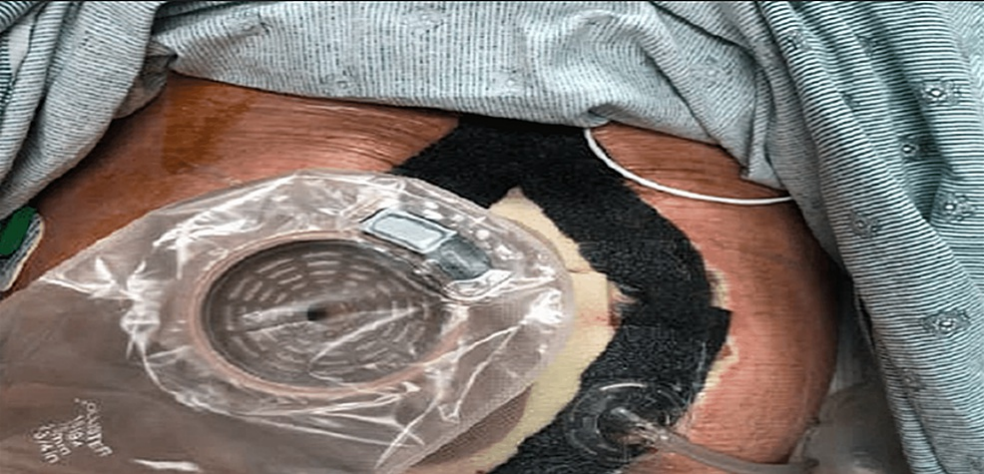
Wound fitted with fistula-VAC appliance. VAC, vacuum assisted closure

Consequently, due to the hostile abdomen, the surgeon determined the best course of action would be to utilize the fistula as an ostomy, with application of a negative pressure wound VAC around the ostomy to promote wound healing and minimize the chance of further bowel leakage (see Figure 3).

**Figure 4:**
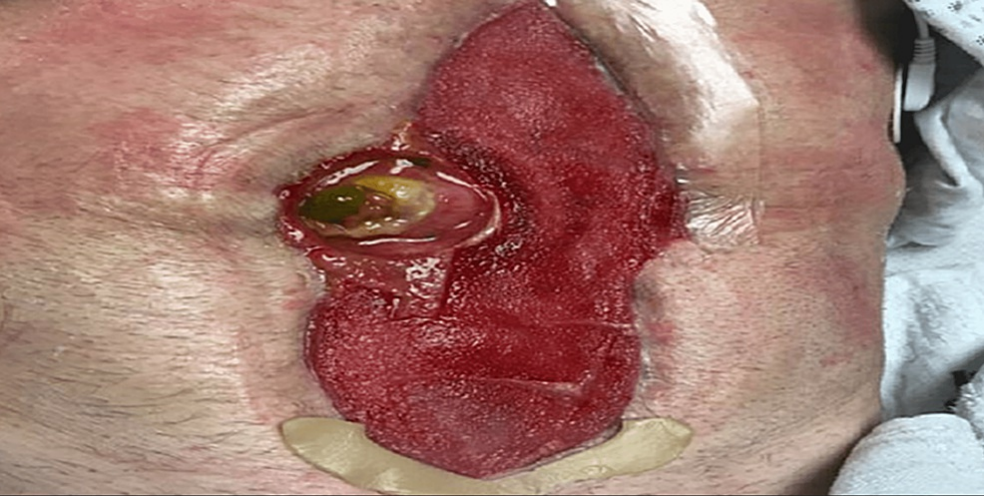
Pre-graft abdominal wound. Approximate wound dimensions: 21 cm x 7 cm.

The VAC appliance consisted of a thin white sponge placed over the entirety of the wound, with a thicker black sponge fitted along the borders. An adhesive film was applied over the top of the sponges to allow for a complete seal and the vacuum pump was connected via tubing that punctured the left lateral aspect of the dressing. Initially, the fistula was fitted with an ostomy paste cone to maintain patency of the stoma. The ostomy paste cone was eventually updated to a more stable, formal stoma appliance, which allowed for easier VAC and ostomy bag changes. The patient had no major complications following VAC placement and was discharged to an inpatient rehabilitation facility on hospital day 45. Approximately one month after the patient’s discharge, he was readmitted with fever, leukocytosis, and right upper quadrant pain. He was found to have a 2 cm x 3 cm fluid accumulation adjacent to the remaining portion right hepatic lobe, which was determined to be related to the biliary stent placed on previous admission. The patient underwent interventional radiology aspiration of the fluid collection, with removal of the stent, and his symptoms resolved. During this admission, the abdominal wound was examined, and it was determined that the wound was suitable for skin grafting (see Figure 4).

**Figure 5:**
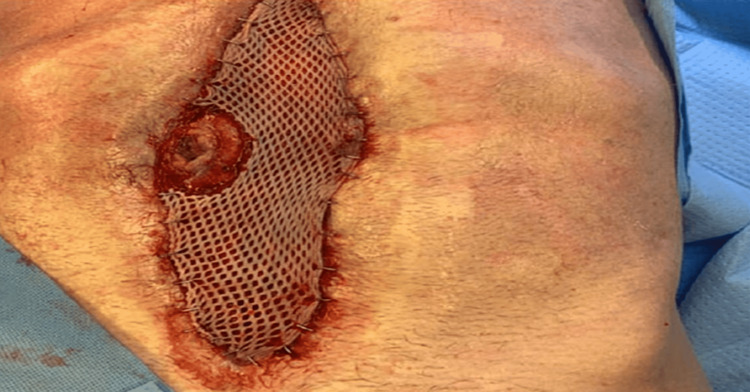
Abdominal wound grafted with a split thickness skin graft, meshed in a 1:1.5 ratio.

The dimensions of the wound had decreased from approximately 25 cm x 15 cm to 21 cm x 7 cm due to the consistent utilization of the negative pressure wound VAC. On hospital day 10 of the patient’s readmission, excisional debridement of the abdominal wound was performed using VERSAJET (Smith & Nephew, St Petersburg, FL) therapy, a split-thickness autograft was harvested from the patient's left lateral thigh, and the graft was meshed in a 1:1.5 ratio and secured over the wound (see Figure 5).

**Figure 6:**
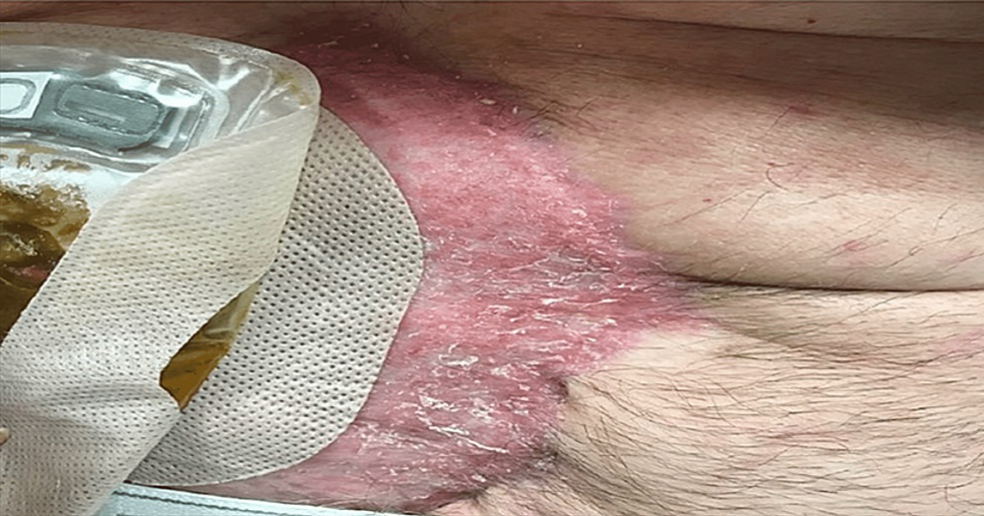
At 13 weeks post-discharge, fistula in place without leakage.

The fistula was maintained and fitted with an ostomy appliance, and the grafted area was dressed in a negative pressure wound VAC. After approximately two more weeks of inpatient treatment following the grafting procedure, the skin graft was successfully taken and the patient was able to tolerate consistent ostomy and wound VAC changes. He was discharged on hospital day 24 to a skilled nursing facility for continued rehabilitation. Thirteen weeks after discharge, the patient was seen in clinic with intestinal fistula in place without any leakage (see Figure 6).

## Discussion

The EAF with OA is very challenging to manage due to the associated high risk of morbidity and mortality [10]. As seen in this patient, EAFs are often complicated by a frozen abdomen which limits the treatment approach since re-entry of the abdomen is likely to cause further damage [2, 7]. There are numerous management techniques outlined in the literature, all with the goal of diversion and collection of the fistula contents, protecting the granulated bowel from further breakdown as well as infection and nutrition issues [1].The technique utilized for this patient was most similar to the “fistula-VAC” technique that is described in numerous pieces of literature [13, 15]. The ability to use the fistula VAC technique is primarily dependent on how easily the fistula can be manipulated and maintained using traditional stoma appliances. In this patient, the location of fistula at the right lateral margin of the open abdominal wound permitted isolation of the stoma at the edge of the wound, without requiring placement of the VAC around the entirety of the opening. This likely would have resulted in closure of the fistula and an increased chance of bowel content leakage. The care team closely monitored the contents of the ostomy bag, cleanliness of the wound edges and VAC sponge, and the general condition of the patient’s skin and granulated viscera. Careful attention to the patient's condition allowed for swift intervention in the event the ostomy bag or wound VAC was compromised, or leakage was observed. Consistent ostomy and VAC sponge changes were an important aspect of keeping the patient’s wound clean, promoting healing and reducing infection risk. The patient’s ostomy bag was changed when full, and the VAC sponge was changed every two days or when cleanliness necessitated changing. Isolation of the fistula also continued to improve as the wound edges approximated with use of the wound VAC. The fistula VAC technique was further utilized after the wound was grafted to promote healing of the graft while continuing to divert bowel contents from the wound. In this patient, fistula-VAC was an effective way to promote wound healing while reducing the chance of complications related to bowel content leakage pre- and post-grafting of the OA.

## Conclusions

Based on the outcome of this patient, fistula-VAC seems to be an effective method for managing an EAF, while also allowing for the eventual closure of the OA. Intestinal contents were effectively contained by the basic ostomy appliance, and use of the negative pressure wound VAC over the course of treatment promoted wound healing and approximation of the wound edges that ultimately permitted definitive grafting of the abdominal wall.
